# Biographical Dictionary of Deceased American Physicians

**Published:** 1867-03

**Authors:** Joseph M. Toner

**Affiliations:** No. 350 Pennsylvania Avenue, Washington, D. C.


### Biographical Dictionary of Deceased American Physicians.

Washington, March 2, 1867.

To American Physicians:—Your attention is respectfully invited to the subjoined inquiries, which are sent to you in furtherance of a plan of collecting material for the publication of a Biographical Dictionary of Deceased American Physicians. The work is already in a state of considerable forwardness, and is designed to contain a biographical sketch of every deceased practitioner of regular medicine, from the earliest settlement of our country to the present time.

Hundreds of physicians have died in different parts of the United States, after devoting a long and useful life to their profession, of whose existence and labors there is no record. This should not be so. The want of a work furnishing an outline of the lives of American physicians has long been felt; and that the project meets the approbation of the profession and of the relatives of deceased medical men, has already been evinced by valuable contributions from different parts of the country.

The success of the undertaking must obviously depend, in a great measure, upon the coöperation of the medical profession, and such relatives and acquaintances of deceased physicians as may have it in their power to communicate the essential information.

The undersigned confidently solicits your aid in furnishing such facts as your knowledge may afford, and answering the following inquiries:

1—Name in full, including first, middle, and last.
2—Place of birth, mentioning town, county, and State.
3—Day, month, and yearth of birth.
4—Full name of father; his occupation and residence.
5—Maiden name of mother, and her father's name in full.
6—Full names of ancesters, near and remote, in regular order.
7—Family matters of interest worthy of record.
8—Teachers, schools and colleges, other than professional, giving dates as far as possible.

9—Medical education, naming teachers, college, with dates of commencing study, and of graduation. Subject of Thesis.
10—Date of commencing practice, and where.
11—Place or places of subsequent residence, with dates.
12—Particulars in regard to practice.
13—Publications, professional and other; their exact titles, in full, with dates and places of publication.
14—Offices held, professional and other, with dates of tenure. Professorships in colleges. Membership in societies. Honorary degrees.
15—Health, habits, religious character, church membership.
16—Date of commencement of last illness, with particulars in regard to same.
17—Date and place of death, and age.
18—Place of interment, and monumental inscription.
19—Full name(s) of wife(ves); her (their) fathers' and mothers' name(s); date(s) of marriage; children, giving the name of each.
20—Any particulars not coming within the above—property accumulated, personal appearance, copy of or reference to any biographical sketch, or obituary notice, in professional or secular prints. Engraving and autograph desired, and, if obtainable, any specimens of writing, published or unpublished. If there be any likeness, mention the artist and possessor. Omit nothing which will give interest to a biographical sketch.
21—Name of informant, and sources of information.

JOSEPH M. TONER, M. D.,
No. 350 Pennsylvania Avenue, Washington, D. C.

